# How conspecific and allospecific eggs and larvae drive oviposition preference in *Drosophila*

**DOI:** 10.1093/chemse/bjae012

**Published:** 2024-04-12

**Authors:** Rolando D Moreira-Soto, Mohammed A Khallaf, Bill S Hansson, Markus Knaden

**Affiliations:** Department of Evolutionary Neuroethology, Max-Planck Institute for Chemical Ecology, Jena, Germany; Universidad de Costa Rica, Centro de Investigación en Enfermedades Tropicales, Facultad de Microbiología, San José, Costa Rica; Department of Evolutionary Neuroethology, Max-Planck Institute for Chemical Ecology, Jena, Germany; Department of Evolutionary Neuroethology, Max-Planck Institute for Chemical Ecology, Jena, Germany; Department of Evolutionary Neuroethology, Max-Planck Institute for Chemical Ecology, Jena, Germany

**Keywords:** egg odors, larval odors, oviposition choice, larval competition

## Abstract

Where to lay the eggs is a crucial decision for females as it influences the success of their offspring. Female flies prefer to lay eggs on food already occupied and consumed by larvae, which facilitates social feeding, but potentially could also lead to detrimental interactions between species. Whether females can modulate their attraction to cues associated with different species is unknown. Here, we analyzed the chemical profiles of eggs and larvae of 16 *Drosophila* species, and tested whether *Drosophila* flies would be attracted to larvae-treated food or food with eggs from 6 different *Drosophila* species. The chemical analyses revealed that larval profiles from different species are strongly overlapping, while egg profiles exhibit significant species specificity. Correspondingly, female flies preferred to lay eggs where they detected whatever species’ larval cues, while we found a significant oviposition preference only for eggs of some species but not others. Our findings suggest that both larval and egg cues present at a given substrate can drive oviposition preference in female flies.

## Introduction

The decision female insects make regarding where to lay eggs is of critical importance. It has direct consequences for the females’ reproductive fitness because both eggs and larvae are vulnerable to predation and larvae have limited mobility to search for better conditions (Dweck et al. 2013; Liu et al. 2017). The choice of oviposition site can thus affect embryo survival, the performance of the young offspring and their phenotype, and can potentially affect even the survival of the ovipositing female (Resetarits 1996; Refsnider and Janzen 2010). One crucial factor in the decision-making process shared by many female insects is the desire to minimize the risk of predation and competition from conspecifics. This has been shown in some insects where egg survival decreased with higher amounts of eggs deposited on a single host plant, potentially due to resource competition and even cannibalism among emerging larvae (Mitchell 1975; Williams and Gilbert 1981; Refsnider and Janzen 2010).

The “mother-knows-best” hypothesis stipulates that females have indeed evolved to oviposit in places that optimize the survival of the offspring (Soto et al. 2015; Liu et al. 2017). However, research has also shown that females do not always lay their eggs in habitat types that maximize embryo survival (Refsnider and Janzen 2010). It seems that other selection pressures may override the differences in survival of embryos among habitat types. This fact also reveals the intricate nature of oviposition site selection, suggesting that the choice is more complex than selecting sites with the highest likelihood of embryonic survival (Refsnider and Janzen 2010). In *Drosophila*, interspecific differences in oviposition preferences are influenced by various environmental factors such as ambient light (Wogaman and Seiger 1983), host chemistry (Richmond and Gerking 1978; Amlou et al. 1998; Fanara and Hasson 2001), host microbial composition (Hoffmann and Harshman 1985; Oakeshott et al. 1989), host texture (David 1970; Rockwell and Grossfield 1978; Fogleman et al. 1981; Chess and Ringo 1985), and substrate temperature (Fogleman 1979; Schnebel and Grossfield 1986).

Oviposition is a process of complex decision making that involves multiple sensory modalities such as vision, olfaction, proprioception, but also taste (Dweck et al. 2013; Liu et al. 2017). While sensory neurons on the ovipositor have been shown to be involved in the final decision, whether or not to lay an egg (Takamura and Fuyama 1980; Chess and Ringo 1985), other appendages such as proboscis, wings, and legs also present taste receptors with sex-specific responses that may be involved in the decision making (Stocker 1994; Meunier et al. 2000; Chyb 2004; Markow and O’Grady 2008).

In *Drosophila* flies, the importance of the choice is further highlighted in several reports, stating that females are highly selective regarding where to lay the eggs and can withhold egg laying until they find an optimal substrate (Yang et al. 2008; Joseph et al. 2009; Schwartz et al. 2012; Azanchi et al. 2013; Fanara et al. 2023). On the other hand, attraction of female *Drosophila* to oviposition sites already occupied by larvae has been observed, although previously attributed to substrate texture rather than social cues provided by eggs or larvae (del Solar and Palomino 1966; Atkinson 1983; Markow and O’Grady 2008). This phenomenon of communal egg laying has been demonstrated and hypothesized to enhance larvae survival by improving oviposition site quality, by the inoculation of the substrate by the adults with yeasts, acting as a larval food source, and because groups of larvae are better at reducing the hyphal growth of molds that compete for food with the larvae (Wertheim et al. 2002; Trienens et al. 2017; Verschut et al. 2023). However, communal egg laying can also lead to challenges in the form of resource competition, growth constraints, and even cannibalism when resources are exhausted (Etienne et al. 2002; Wertheim et al. 2002; Narasimha et al. 2019; Bailly et al. 2023). The fitness benefits of communal egg laying also depend on the number of larvae developing at the communal site (Wertheim et al. 2002; Trienens et al. 2017). Balance becomes imperative, as too low number of larvae could fail to survive due to the growth of harmful fungi. At the same time, if the density is too high an increased attraction of natural enemies may occur, and high density can also lead to resource competition or cannibalism (Verschut et al. 2023).

When it comes to communal egg laying, it has been hypothesized that females should be more strongly attracted to cues associated with beneficial larval species, genotypes, and densities (Beltramí et al. 2012). The cuticular hydrocarbons (CHCs) of *Drosophila* species are known to be species specific in composition and at least adult compounds are involved in both intra- and interspecific communication (Ferveur 2005; Khallaf et al. 2021; Tungadi et al. 2022, 2023). It, however, remains open, whether CHCs of *Drosophila* eggs and larvae are also species specific, and whether gravid female *Drosophila* respond differentially to the presence of eggs and larvae from different *Drosophila* species. Here we analyze the chemical profiles of the different life stages of several *Drosophila* species and investigate whether the communal egg laying is modulated by species-specific chemical cues of eggs and larvae that are already present in the substrate.

## Results

### Females, and to lower extent eggs, display more species-specific chemical profiles than larvae

**Fig. 1.**
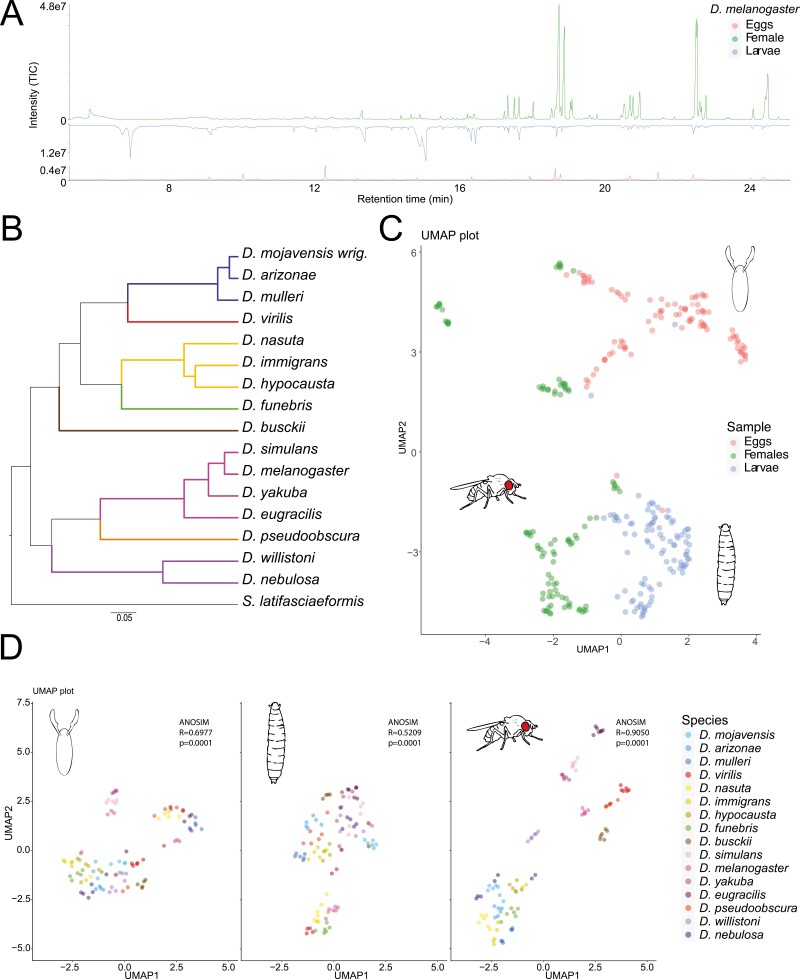
Chemical profiles of eggs, larvae and females from 16 *Drosophila* species. (A) Representative gas chromatography (GC) from eggs, larvae, and female of *D. melanogaster* measured by TDU-GC–MS. (B) Phylogenetic tree of the 16 species analyzed (excluding outgroup). The colors represent the phylogenetic grouping: *melanogaster* group (fuchsia), *willistoni* group (purple), *obscura group* (orange), *immigrans* group (sand), *virilis* group (red), *funebris* group (green), *repleta* group (blue) and *D. busckii* (brown). Scale bar for branch length represents the number of substitutions per site. (C) UMAP showing all species and sample types, each dot represents a single replicate, and there are 6 or more replicates per species. (D) UMAPs of each developmental state separated, with colors representing the different species. Statistical analyses were performed to test the similarity between the species (ANOSIM, 999 permutations).

To determine whether chemical profiles in *Drosophila* are more similar in terms of species or developmental stages, we analyzed the chemical profiles of eggs, larvae, and mated females of 16 *Drosophila* species. These species represent various groups spread across the phylogeny of the *Drosophila* genus, and for each, we conducted 6 or more replicates, resulting in a total of 294 chromatograms analyzed by using thermal desorption unit gas chromatography/mass spectrometry (TDU GC-MS) (Fig. 1A, B; Supplementary Fig. S1). We detected 127 compounds [i.e. features with distinct *m/z* (mass-to-charge ratios)] in eggs, while in larvae and mated females, the number nearly doubled, reaching 277 and 231, respectively.

Comparing all samples in a Uniform Manifold Approximation and Projection (UMAP) resulted in groups of developmental stages, with eggs, larvae, and females of different species grouping together (Fig. 1C). When we, however, analyzed the chemical profiles of eggs, larvae, and females separately, the chemistry of females appeared to be more species specific (ANOSIM, Bray Curtis coefficient *R* = 0.90, *P* = 0.001; 95% CI [0.84, 0.93]) than that of eggs (*R* = 0.7, *P* = 0.001; 95% CI [0.57, 0.76]) and even more than that of larvae (*R* = 0.52, *P* = 0.001; 95% CI [0.4, 0.56]) (Fig. 1D). Many female and certain egg profiles also clustered not only based on species but also on their respective species group, while the clustering of the larvae appeared to be more arbitrary (Fig. 1D; Supplementary Fig. S2).

### Larvae show stronger chemical similarity than eggs and mated females

**Fig. 2.**
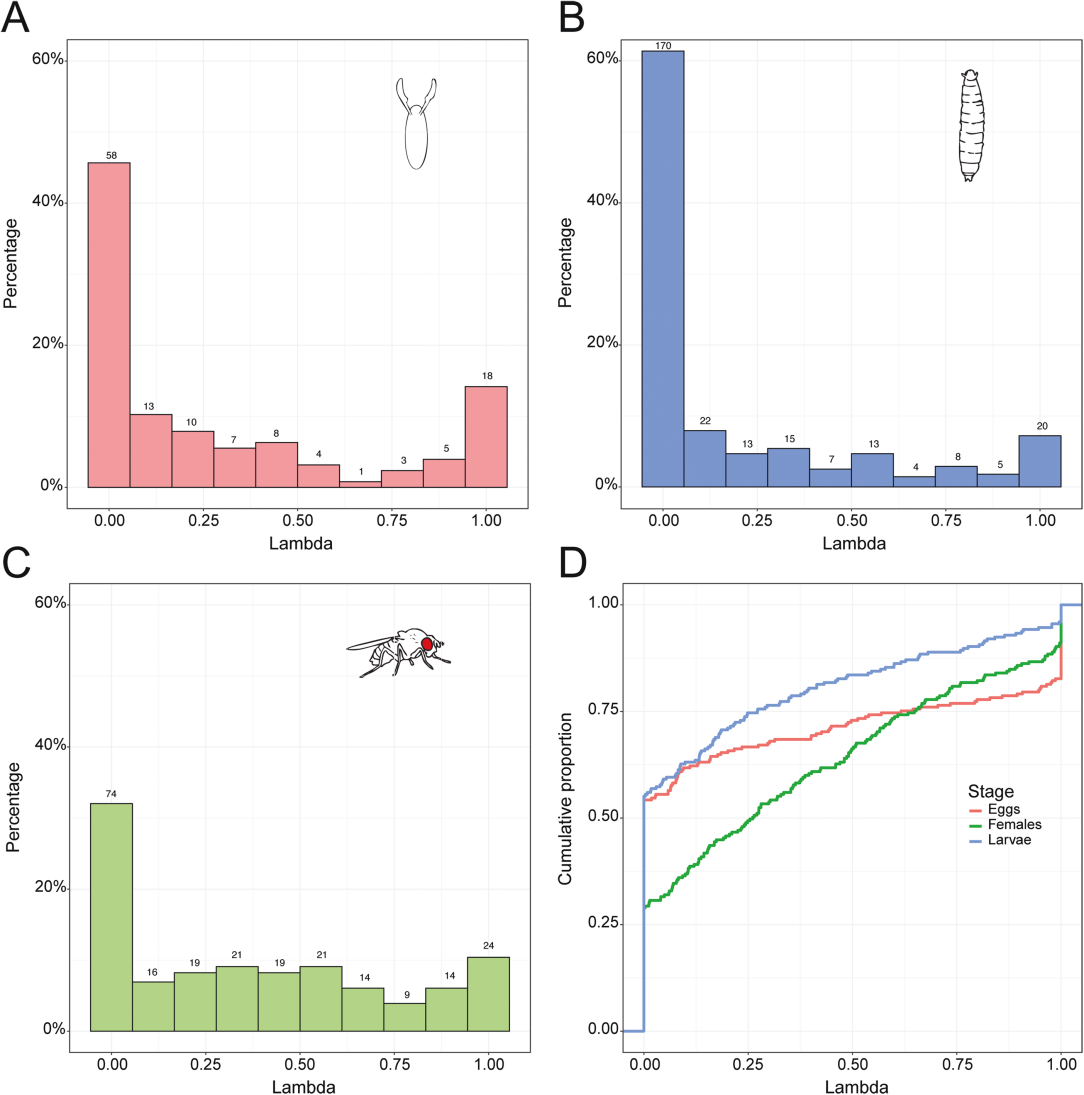
Distributions of Pagel’s λ values for all individual compounds, for eggs, larvae, and females. (A–C) Histograms of the distributions, representing the percentage and number of compounds on each λ value, with values closer to 1 representing a high phylogenetic signal. The numbers over each column represent the number of compounds on each λ value. (D) Cumulative distributions functions for all 3 developmental stages combined.

We next determined the number of compounds that presented a significant difference between any of the species analyzed. From the total of compounds found in eggs, larvae, and females, 20.47%, 8.66%, and 16.45% of the compounds had statistical difference between any of the species (*P* < 0.05 with all *P*-values being corrected for Bonferroni correction). This is in agreement with our additional analysis, where we investigated the phylogenetic signal of the individual compounds. We used Pagel’s λ to measure the statistical dependence among species’ trait values due to their phylogenetic relationships. In this case, Pagel’s λ gives us the phylogenetic signal of all individual compounds, for each of the life stages (eggs, larvae, adult females) analyzed separately. In eggs, 14.17% of the λ values exhibited a high phylogenetic signal between 0.9 and 1, while only 7.22% and 10.39% of the values reached this level in larvae and in females, respectively (Fig. 2). The global λ distributions were statistically different between eggs and females (*D* = 0.18; *P* = 0.014, Kolmogorov–Smirnov two-sample test with *P*-values adjusted for Bonferroni correction for repeated comparisons), as well as larvae and females (*D* = 31; *P* = 8.8 × 10^−11^), but showed only a strong tendency between eggs and larvae (*D* = 0.16; *P* = 0.054). Taken together, our data suggest that larvae from the different species are chemically more similar to each other than in eggs and mated females.

Finally, we investigated whether any of the recently identified male- and female-specific compounds (Khallaf et al. 2021) are transferred to the eggs. From the 16 *Drosophila* species included in our analysis, a total of 24 male- and 9 female-specific compounds have been reported. From those, we found 16 male-specific and 6 female-specific compounds in eggs, which represent 66.6% of the reported male and female compounds (details in Supplementary Table S1, based on Khallaf et al. (2021)). Notably, in *Drosophila*, male-specific compounds are known to be transferred from males to females during copulation (Bartelt et al. 1985; Laturney and Billeter 2016; Khallaf et al. 2021). Our observations reveal that many of them are further passed from females, along with some female-specific compounds, to their eggs during oviposition.

### *Drosophila* females prefer to oviposit on larvae-treated food

**Fig. 3.**
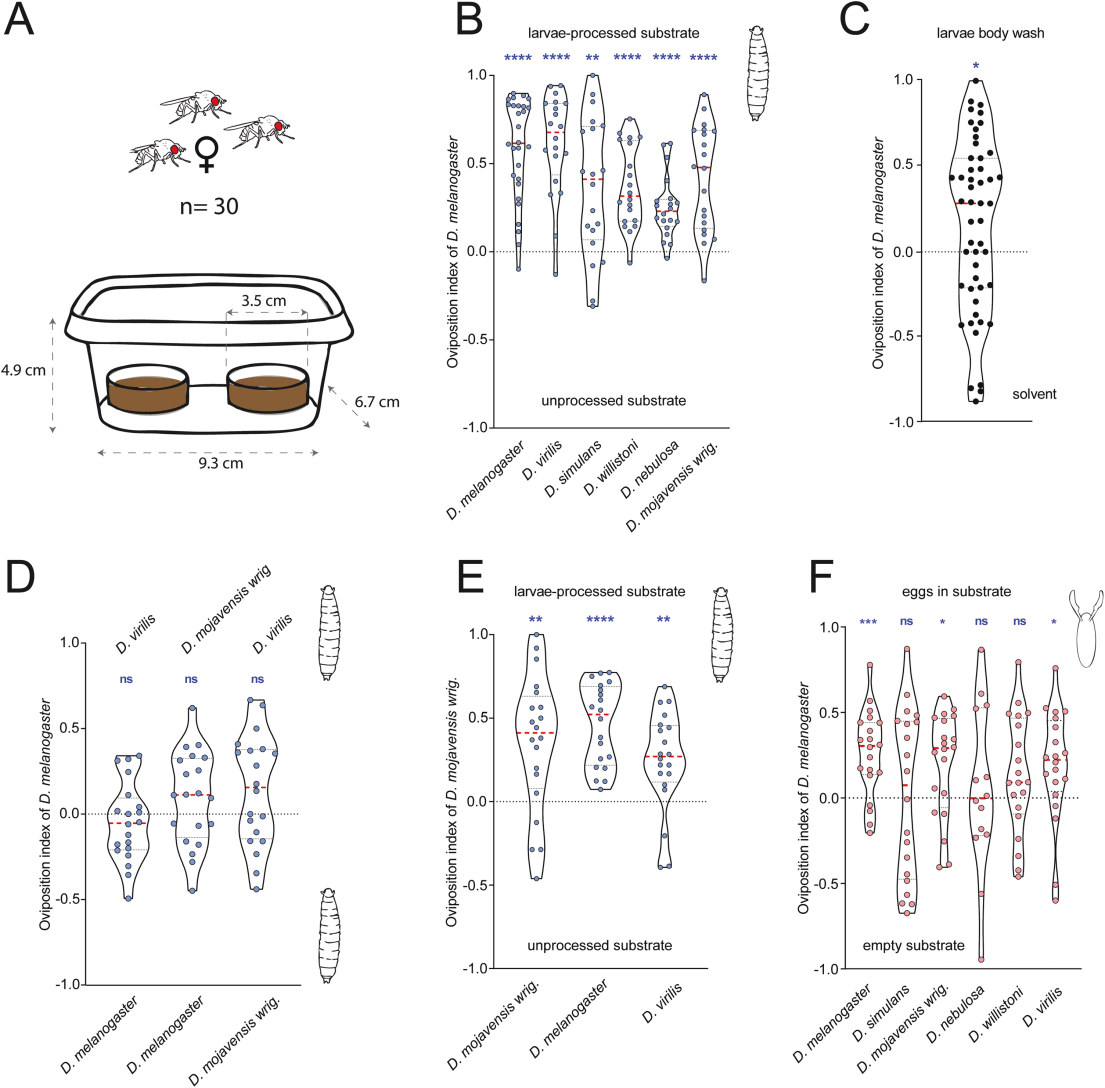
Oviposition experiments in *Drosophila* females (30 per assay). (A) Visual representation of the 2-choice assays performed. (B) Oviposition index of *Drosophila melanogaster* against normal fly food vs food processed by larvae from 6 *Drosophila* species. (C) Oviposition index of *Drosophila melanogaster* females against fly food with conspecific larvae body wash vs fly food with solvent (*n* = 50). (D) Oviposition index of *D. melanogaster* against fly food processed by larvae from 2 *Drosophila* species. (E) *D. mojavensis* wrigleyi oviposition index against untreated food or larvae-processed food from 3 species. (F) Oviposition index of *D. melanogaster* with a choice between normal fly food and food with 10 eggs from 6 *Drosophila* species. In (B–D), the larvae were left 24 h and removed before introducing the gravid females. Stars show statistical significance (*n* ≥ 20, Wilcoxon signed-rank test, *P < 0.05; **P < 0,01; ***P < 0.001, ****P < 0.0001).

Having shown that larvae chemical profiles seem to be less species specific than the ones of females and eggs (Figs. 1 and 2; Supplementary Fig. S2), we wanted to determine if *Drosophila melanogaster* ovipositing females would choose or avoid places where they detect conspecific or heterospecific larvae. For this we tested the preference of *D. melanogaster* females when given the choice between the substrate that was before either processed by larvae of a subset of 6 *Drosophila* species or was unprocessed (Fig. 3A). Gravid *D. melanogaster* females preferred the larvae-processed substrate over the unprocessed substrate, regardless to which species the processing larvae belonged to (Fig. 3B). It seems that the benefit of communal breeding in *D. melanogaster*, regardless of the species-identity of the detected larvae, is higher than any potential danger the larvae might pose. When we performed the same assay, but mimicked the presence of larvae by just adding compounds in the wash from *D. melanogaster* larvae to the substrate, gravid females still exhibited significant preference to the substrate (Fig. 3C), suggesting that indeed the CHCs of the larvae are involved in the female decision making.

Having shown that larval cues from all species were attractive to gravid *D. melanogaster* females, we next asked whether flies still prefer one species over the other. We, therefore, tested *D. melanogaster* females with a choice between 2 substrates, both were treated before either by *D. melanogaster* larvae or by larvae of *D. virilis*, or *D. mojavensis wrigleyi*. In none of the 3 binary combinations tested, females showed any oviposition preference (Fig. 3D). Evidently, *D. melanogaster* females do not exhibit a preference based on the species of larvae already present at a particular oviposition site.

Is this rather unspecific preference for larval-treated food restricted to females of *D. melanogaster* or do *Drosophila* females of other species exhibit a similar oviposition behavior? To answer this question, we tested *D. mojavensis wrigleyi* females with the same 2-choice oviposition assay, using untreated substrate vs larvae-processed substrate from 3 different *Drosophila* species. Similar to *D. melanogaster* females, *D. mojavensis wrigleyi* always preferred the source that was treated with larvae before, regardless of whether these larvae were conspecifics or not (Fig. 3E).

### *D. melanogaster* females prefer to lay eggs alongside eggs of some species

To determine whether eggs of different *Drosophila* species would attract ovipositing females as seen with larvae-treated food, we tested *D. melanogaster* females again in a 2-choice oviposition assay. This time, the flies had to choose between substrate with or without eggs of one of the 6 above-mentioned *Drosophila* species. *D. melanogaster* females preferentially oviposited on food containing eggs of *D. melanogaster*, *D. virilis*, and *D. mojavensis wrigleyi*. For food containing eggs of *D. simulans*, *D. nebulosa*, and *D. willistoni,* we did not detect a significant oviposition preference, but we do not rule out that there may be some preference that we lack the statistical power to detect. Due to high inter-trial variance, the oviposition indices did not differ significantly among the foods containing the eggs of different species (Kruskal–Wallis test, *P* = 0.31). These data suggest that eggs of certain species attract females to oviposit, while it is possible that eggs of other species do not affect the oviposition choice (Fig. 3F).

## Discussion

Drosophilid flies are known to exhibit species-specific chemical cues which they use to communicate intra- and interspecifically (Antony et al. 1985; Bartelt et al. 1985; Ferveur 2005; Yew et al. 2009; Laturney and Billeter 2016; Khallaf et al. 2021; Tungadi et al. 2023). Here we described the chemistry of eggs, larvae, and mated females from 16 *Drosophila* species, and we found that the chemical profiles across the larvae species tested is more similar, i.e. less species-specific than chemistry in eggs or mated females (Figs. 1D and 2). Correspondingly, in our oviposition assay *D. melanogaster* and *D. mojavensis wrigleyi* females preferred to oviposit where they detected *Drosophila* larvae, independent of the species the larvae belonged to (Fig. 3). This agrees with past reports stating that *D. melanogaster* prefer to oviposit communally, suggesting that larvae may benefit from forming social foraging groups with an improved ability to dig into the substrate (Durisko, Kemp, et al. 2014). The group foraging strategy brings several advantages: first, it allows larvae to get inside the fruit quicker, where the temperature and humidity are much less variable than at the surface, and where larvae might be better protected from parasitoids; second, larval burrowing may serve to break down and soften food, making it easier to ingest; finally, the digging of the larvae also may stir the food substrate, which can prevent competitive mold growth, and can facilitate the growth of beneficial yeast species (Bakula 1969; Rohlfs 2005a, 2005b; Stamps et al. 2012). In the case of *Drosophila melanogaster*, it was shown recently that females cloak their eggs with pheromones that protect their eggs from cannibalism, deterring larvae from consuming the eggs (Narasimha et al. 2019). This could mean that females do not care of the larvae species present on the food, since the eggs have this protective strategy.

It was already known that both male and female *D. melanogaster* flies are attracted to odors emanating from food that has been occupied by conspecific larvae (Durisko and Dukas 2013; Durisko, Anderson et al. 2014). The present larvae and their feces seem to be an indicator for adults that a substrate is nutritionally sufficient (Durisko, Kemp et al. 2014; Golden and Dukas 2014). Our findings suggest that this preference is not restricted to *D. melanogaster* and its larvae. Moreover, other *Drosophila* species also prefer larvae-treated food and this preference does not seem to be restricted to conspecific larvae. On the other hand, we tested *D. melanogaster* females in an assay using larvae body wash, and the preference to oviposit in the side with larvae cues was still there (Fig. 3C), but was not as significant as larvae-processed food (Fig. 3B). This indicates that the cues attracting the flies to the larvae-treated food are not only chemicals from the surface of the larvae, but also other signals left on the food, for example feces and the corresponding microbes therein.

Ovipositing flies are known to inoculate food with microbes (living on the egg shells) which then populate the food and themselves become a resource for growing fly larvae (Bakula 1969). These microbes are known to guide fruit flies to food with a favorable microbial environment, as the larvae microbiome could suppress hostile microbes (Venu et al. 2014). *Drosophila*-associated microbes are known to catabolize predominantly sucrose from fruit, resulting in depleted sucrose and enriched bacteria, i.e. an ecological niche that is obviously preferred by ovipositing *Drosophila* (Liu et al. 2017). However, it remains uncertain whether any of the tested fly species carry and transfer species-specific microbes in their gut (and whether this species specificity remains also after the flies have been kept for many generations in the lab). Future tests with freshly captured flies shall hence reveal, whether the indifference of female flies, when they must choose between food treated by conspecific or heterospecific larvae, is partially due to a potential homogenizing effect of breeding the flies since generations in the lab. Anyhow, the former presence of larvae, regardless of which species, seems to be a strong cue governing female oviposition attraction.

On the other hand, it is known that interactions between different larval species can affect larval development and survival differently, slowing down in some cases the larval developmental rates (Budnik and Brncic 1974). It was therefore expected that female flies would not be attracted by larvae from some *Drosophila* species, as some species combinations could be beneficial and others not (Durisko, Anderson et al. 2014). Studies have shown in the case of *Drosophila suzukii* that females are deterred to oviposit by the presence of *D. melanogaster* larvae, contrary to the behavior of the species we tested, which suggests that *D. suzukii* perceives *D. melanogaster* as a competitor species, modulating the oviposition choice (Tungadi et al. 2022, 2023). Furthermore, our results suggest that *D. melanogaster* and *D. mojavensis* females do not distinguish between larvae of different species. Apparently, either the advantage given by the communal egg laying is so significant for these species that females just ignore potential negative interactions with other larvae species, or these *Drosophila* females cannot distinguish the larvae species based on the cues left on the food. The latter would be in agreement with our findings that the species specificity of chemical profiles of larvae is rather low (Fig. 1D). Related to this, larvae also showed a lower percentage of compounds with a Pagel’s λ closer to 1, which suggests that the chemical profiles in the larvae seem to be less related to the phylogeny than in eggs or females (Fig. 2). This can also mean that a higher percentage of chemical compounds present in larvae is more correlated with ecological niche than phylogenetic distance. Some traits in *Drosophila* show a higher correlation to the ecological niche than to phylogenetic distance, as shown in the case of projection neuron—Kenyon cell connectivity in some *Drosophila* species (Ellis et al., 2023). However, the correlation of the chemical profiles to the ecological niche would need to be further studied.

Interestingly, we found the presence of male- and female-specific compounds in eggs. Many male-specific compounds are known to be transferred to females during mating (Bartelt et al. 1985; Laturney and Billeter 2016; Khallaf et al. 2021). Pheromones are also known to be present in the reproductive tract of mated females, and some compounds seem to come from male ejaculate like cVA, which is produced in the male’s ejaculatory bulb (Guiraudie-Capraz et al. 2007; Laturney and Billeter 2016; Narasimha et al. 2019). Therefore, it is likely that male compounds get also transferred from the females to the eggs during oviposition. The egg wax-layer synthesis is likely to involve transportation of maternal and paternal hydrocarbons from the oenocytes and deposited seminal fluid, respectively, to the ovary during oogenesis (Narasimha et al. 2019). Thus, it seems that in the *Drosophila* genus frequently both parents contribute toward provisioning the pheromonal content of the egg wax layer. Some of the compounds in the wax layer of eggs turned out to be identical to aggregation pheromones already known to be deposited by adult male and female flies (Ferveur 2005; Wertheim et al. 2006; Narasimha et al. 2019).

It was therefore not unexpected that, in our assays, females preferred substrates with eggs over substrates without. Interestingly, however, while eggs of some species attracted ovipositing *D. melanogaster* females, we did not detect any significant oviposition preference toward food containing eggs of other species. A contrasting behavior was reported in *D. suzukii* females, where the oviposition was tested using conspecific or allospecific eggs, and females avoid some species while showing no preference with others, including conspecifics (Kidera and Takahashi, 2020). Interestingly, *D. suzukii* females did not show attraction rather avoidance against some competitor species. Results from Kidera and Takahashi (2020) showed that flies were able to discriminate eggs from different species. In our case, even though we did not observe any significant difference in the oviposition indices for foods containing eggs of different species, it is intriguing that we only detected significant oviposition preferences for eggs of some species, not others. The presence of male- and female-specific compounds found in eggs could potentially influence these decisions, but the choice between food containing eggs of one species vs food containing eggs of another would need to be further tested.

Interestingly, eggs of *D. melanogaster* were attractive while we did not detect a significant oviposition preference for eggs of *D. simulans* for ovipositing *D. melanogaster* females, even though eggs of these 2 species seem to be close in terms of chemical profiles (Fig. 1D; Supplementary Fig. S2). However, it is not unexpected since the UMAP shows the overall structure of the data, but differences in a few but behaviorally relevant chemical compounds could be overlooked. We found, for example, the male-specific (*Z*)-7-pentacosene and (*Z*)-7-tricosene and the female-specific (*Z*,*Z*)-7,11-pentacosadiene, (*Z*,*Z*)-7,11-heptacosadiene, and (*Z*,*Z*)-7,11-nonacosadiene in eggs of *D. melanogaster*, but not in those of *D. simulans* (Supplementary Table S1). Even if we do not have enough behavioral evidence to prove that females are able to distinguish the eggs of different species present in the food, our results from the chemistry analysis of eggs from 16 *Drosophila* species found significant species specificity. The attraction to eggs of some species, but the lack of significant attraction to eggs of other species, could also suggest that *Drosophila* flies need to make this distinction between species at an early stage, i.e. in the presence of eggs. However, when larvae are present at a later stage the distinction is not necessary or possible. Reports of cannibalism in *D. melanogaster* state that young larvae can predate on third instar larvae (Ahmad et al. 2015), i.e. contrary to the expectation, young larvae might represent a higher danger. This could therefore mean that the presence of first or second instar larvae in the food might result in differences in attraction, unlike what we found with 3rd instar larvae, but this would need to be tested further.

Taken together, our results show that *Drosophila* females are attracted to oviposit where they detect larvae, independent of the species, but in the case of eggs, they seem to be attracted to some species, but not to others. This brings us back to the question on whether females modulate their attraction to oviposit with beneficial species, and our results suggest that the females do not use larval cues, but they may be using egg (or female-transferred) cues to decide where to oviposit. Our chemical analyses support this hypothesis, as the larvae of different species seem to be more similar in terms of chemical profiles, while the eggs have more species specificity. The presence of male- and female-specific compounds in eggs suggests their possible role in serving as signals for oviposition decisions. Further work is, however, needed to elucidate the individual cues oviposition decisions rely on, and the neurobiological pathways that modulate this behavior.

## Methods

### Fly stocks

The study utilized wild-type flies that were acquired from the National Drosophila Species Stock Centre (NDSSC; http://blogs.cornell.edu/drosophila/) and the Kyoto Stock Center (Kyoto DGGR; https://kyotofly.kit.jp/cgi-bin/stocks/index.cgi). Stock numbers and breeding diets are listed in Supplementary Table S2. The flies were raised under specific conditions: a temperature of 25 °C, a 12-h light and 12-h dark cycle, and 70% relative humidity. For more information about the food recipe, refer to the Drosophila Species Stock Centre (http://blogs.cornell.edu/drosophila/recipes/). The care and treatment of all flies adhered to applicable ethical regulations.

### Chemical analyses and phylogenetics

#### Thermal desorption-gas chromatography-mass spectrometry

To obtain the chemical profiles of mated females, eggs, and larvae, we selected 16 species spread across the *Drosophila* phylogeny. Individual 10-day-old female mated flies were decapitated to avoid them from escaping, placed in standard microvials in thermal desorption tubes and transferred into a GERSTEL thermal desorption unit (www.gerstel.de) using a GERSTEL MPS 2 XL multipurpose sampler. In the case of eggs, we placed 5 eggs inside the standard microvials for each analysis. For larvae, a third instar larva was placed inside the microvials and 2 µl of hexane were added to avoid larvae from escaping. From all species and developmental stages, we analyzed at least 6 replicates, yielding a total of 294 individual analysis.

In terms of the gas chromatography-mass spectrometry (GC-MS) device, we used an Agilent GC 7890 A fitted with an MS 5975 C inert XL MSD unit (www.agilent.com), equipped with an HP5-MS UI column (19091S-433UI; Agilent Technologies). After desorption at 250 °C for 8 min, the volatiles were trapped at − 50 °C using liquid nitrogen for cooling. To transfer the components to the GC column, the vaporizer injector was heated gradually to 270 °C (12 °C/s) and held for 5 min. The temperature of the GC oven was held at 50 °C for 3 min, gradually increased (15 °C/min) to 250 °C and held for 3 min, and then to 280 °C (20 °C/min) and held for 20 min. For MS, the transfer line, source, and quad were held at 270, 230, and 150 °C, respectively.

The raw GC-MS data were exported to AIA format using MSD ChemStation (Agilent Technologies). The exported files were loaded into R (4.1.0) and the XCMS package was used for peak detection and retention time alignment (Smith et al. 2006). In XCMS, the centWave algorithm was used for peak detection using the following parameters: ∆*m*/*z* of 30 ppm, minimum peak width of 3 s, maximum peak width of 50 s, and signal-to-noise threshold of 20. Retention time correction was performed using the obiwarp function, and for the grouping, an *m*/*z* width of 0.1, base width of 5, and minimum fraction of 0.1 were used. All chromatographic peaks before 540 s and after 1980 s were excluded. This analysis was done for eggs, larvae, and mated females separately, and with all samples together.

The XCMS data (intensities of compounds, i.e. features with distinct *m/z* (mass-to-charge ratios)) were normalized by the sum of all features per sample. From this, samples were compared using a UMAP in R (4.1.0) with umap package (McInnes et al. 2020). To test the similarity of the chemical profiles in different species, an analysis of similarity (ANOSIM) was performed with the vegan R package (Oksanen et al. 2022), using the Bray–Curtis coefficient, for each developmental state separately. For the ANOSIM, a 95% confidence interval of the R values was obtained with a bootstrap of 1000 using the R package boot (Canty and Ripley 2022). We also tested for statistical difference in the abundance from all compounds found in eggs, larvae, and females, with Tukey’s test for multiple comparisons of means (*P* < 0.05 with all *P*-values being corrected for Bonferroni correction), using GraphPad Prism v. 9 (https://www.graphpad.com).

### Phylogeny and estimation of phylogenetic signals Pagel’s λ

Our analysis was based on the phylogeny from Khallaf et al. (2021). Briefly, the orthologous protein-coding sequences were extracted from genomes or pseudogenomes using genomic features (GFF) from reference species. Sequences were aligned by codon using TranslatorX and cleaned with GBlocks. Aligned sequences were concatenated for each species, and a maximum likelihood tree was inferred with a bootstrap of 100 using RAxML 8.2.4. Finally branch lengths were optimized using ForeSeqs (see Khallaf et al. (2021) for details). The tree was loaded into R (4.1.0) and edited using ape package (Paradis et al. 2004).

The phylogenetic signals contained in each chemical component were estimated by combining the normalized peak intensity with the phylogeny, using the phylosig function in the phytools R package (Revell 2012). The Pagel’s λ was calculated for eggs, larvae, and females separately. The histograms and cumulative distribution functions of the Pagel’s lambda values were obtained in R (4.1.0) with the package ggplot2 (Wickham, 2016), and a Kolmogorov–Smirnov test was done in R (4.1.0) to test differences between the distributions.

### Behavioral experiments

#### Oviposition assays

To assess the behavioral response of females to larvae and egg cues, we selected a subset of 6 *Drosophila* species spread across the phylogeny. We tested groups of 30 *D. melanogaster* gravid females (8 to 10 days old) in a 2-choice assay. In a transparent salad box (9.3 cm × 6.7 cm × 4.9 cm) they could chose during 24 h to oviposit on a petri dish (diameter, 3.5 cm) containing normal fly food (control) or on an identical petri dish that contained fly food which was either processed by larvae before or contained eggs (Fig. 3A). In the lids of the boxes, we made 20 small holes to favor air flow. For each treatment, a minimum of 20 replicates were done. All behavioral experiments were performed under normal white light at 25 °C and 70% humidity.

To test for oviposition using larvae cues, we treated fly food with 5 larvae of each species. Third instar larvae were left on the food for 24 h and then removed before testing the oviposition choice of *D. melanogaster* females. To avoid any mechanical cues, control food without larvae was manually processed accordingly. To see whether the results were similar for other *Drosophila* species, we also tested *D. mojavensis* wrigleyi females under the same conditions. Finally, we tested *D. melanogaster* females with a choice between food processed by larvae of 2 different *Drosophila* species.

To test the oviposition behavior in *D. melanogaster* females against egg cues, we tested the gravid females in the same 2-choice assay, using the same 6 species tested for larvae cues, but the choice was between normal fly food and fly food with 10 *Drosophila* eggs.

To assess the preference for oviposition, we quantified the number of eggs present on each side of the 2-choice assay. Subsequently, we calculated an oviposition index (OI): [OI = (number of eggs laid on the experimental food − number of eggs laid on the control food)/ total number of eggs laid]. To statistically test whether the OIs were significantly different from zero, Wilcoxon signed-rank tests were conducted, and to test the difference between oviposition indices, a Kruskal–Wallis test was done, using GraphPad Prism v. 9. (https://www.graphpad.com).

### Oviposition assay using larvae body wash

We wanted to isolate the effect of the surface chemicals from the larvae in the oviposition choice, so we repeated the 2-choice assay but instead of larvae-treated food we used food containing larvae body wash. For this, we collected larvae in a 2 ml glass vial and used 5 µl of dichlormethane per larvae. From this body wash, we placed 15 µl in a circle of filter paper (diameter of 1 cm) in the center of the fly food plate, and in the other plate the filter paper contained dichlormethane. The eggs were counted, the OI was calculated, and the statistical significance was calculated as before.

## Supplementary Material

bjae012_suppl_Supplementary_Figures_S1

bjae012_suppl_Supplementary_Figures_S2

bjae012_suppl_Supplementary_Tables_S1

bjae012_suppl_Supplementary_Tables_S2

## Data Availability

The data underlying this article are available in the article and in its online supplementary material.
