# Supplementary figures and images for: How conspecific and allospecific eggs and larvae drive oviposition preference in Drosophila
Source: Chem Senses. 2024 Apr 12;49:bjae012. doi: 10.1093/chemse/bjae012 (PMC11017781; doi:10.1093/chemse/bjae012)

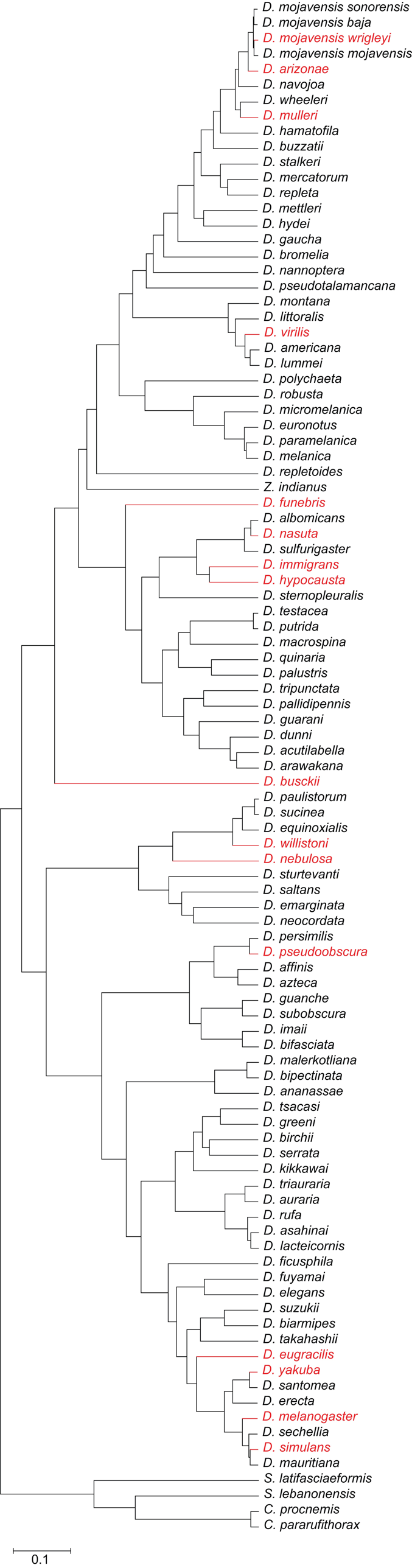

Supplement: bjae012_suppl_Supplementary_Figures_S1 [file bjae012_suppl_supplementary_figures_s1.jpeg]

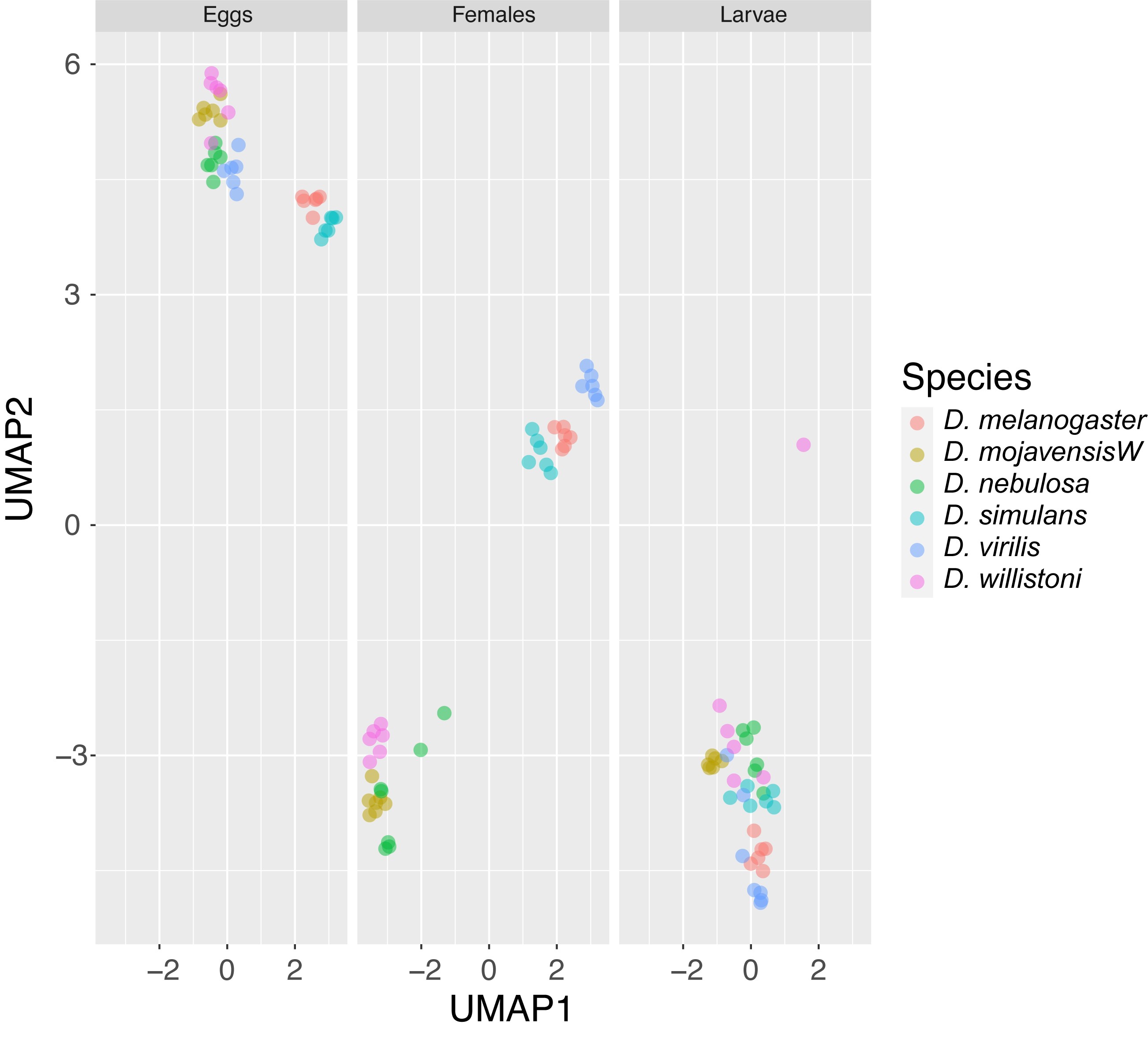

Supplement: bjae012_suppl_Supplementary_Figures_S2 [file bjae012_suppl_supplementary_figures_s2.jpeg]
